# Thermophilic aerobic digestion using aquaculture sludge from rainbow trout aquaculture facilities: effect of salinity

**DOI:** 10.3389/fmicb.2024.1488041

**Published:** 2024-11-06

**Authors:** Jihyun Chun, Su Min Kim, Gwangil Ko, Hyo Jeong Shin, Minjae Kim, Hyun Uk Cho

**Affiliations:** ^1^Department of Marine Environmental Engineering, Gyeongsang National University, Tongyeong, Gyeongnam, Republic of Korea; ^2^Department of Chemical Engineering, Pohang University of Science and Technology, Pohang, Gyeongbuk, Republic of Korea; ^3^Civil Engineering, University of Kentucky, Lexington, KY, United States

**Keywords:** aquaculture sludge, thermophilic aerobic digestion, microbial community, salinity, biological pretreatment

## Abstract

The objectives of this study were to evaluate the potential of using thermophilic aerobic digestion (TAD) to hydrolyze aquaculture sludge, and to investigate the hydrolysis efficiency and changes in microbial community structure during TAD at 0, 15, and 30 practical salinity units (psu). As digestion progressed, soluble organic matter concentrations in all reactors increased to their maximum values at 6 h. The hydrolysis efficiency at 6 h decreased as salinity increased: 2.42% at 0 psu, 1.78% at 15 psu, and 1.04% at 30 psu. The microbial community compositions at the genus level prominently differed in the relative abundances of dominant bacteria between 0 psu and 30 psu. The relative abundance of genera *Iodidimonas* and *Tepidiphilus* increased significantly as salinity increased. Increase in the salinity at which thermophilic aerobic digestion of aquaculture sludge was conducted altered the microbial community structure, which in turn decreased the efficiency of organic matter hydrolysis.

## 1 Introduction

Aquaculture in South Korea has increased by almost 300% over the past 30 years (Park et al., 2021). This growth has been accompanied by an increase in the quantity of organic wastes generated from aquaculture facilities, and in the concerns about environmental pollution near them. Sludge discharged from fish farming systems contains fish feces, uneaten feed, and other metabolites, which are rich in biodegradable organic matter such as proteins and lipids, as well as in other nitrogen compounds. Without proper treatment, the discharge of these substances into nearby water bodies can lead to eutrophication and adversely affect the biodiversity of aquatic ecosystems (Galintin et al., 2021; Lunda et al., 2019).

Anaerobic digestion has been widely used to treat various organic solid wastes due to its ability to reduce organic solids while recovering biogases such as CH_4_ and H_2_ (Liu et al., 2023; Ding et al., 2023; Lee et al., 2024). During anaerobic digestion, the hydrolysis of organic solid waste is the primary rate-limiting step; increasing the solubilization of organic matter in the substrate can increase the yields of CH_4_ and H_2_ (Krohn et al., 2022; Li X. et al., 2020). Therefore, use of thermophilic aerobic digestion (TAD) as a biological pretreatment technique can increase the hydrolysis of organic solid waste and boost biogas production when the pretreated waste is used in subsequent anaerobic digestion (Zhang A. et al., 2022; Jang et al., 2018).

TAD facilitates breakdown of organic solid waste by releasing proteases from thermophilic aerobic microorganisms during the digestion process, leading to a relatively rapid reduction in volatile suspended solids (VSS) compared to anaerobic digestion. TAD also significantly increases the concentration of soluble organic products, such as solubilized proteins, carbohydrates, and volatile fatty acids (VFAs) (Jang et al., 2013). Moreover, TAD operates at high temperatures, and therefore effectively inactivates pathogenic microorganisms within the organic solid waste. Furthermore, self-heating due to the metabolic activities of thermophilic aerobic microorganisms can reduce operational costs. Due to these advantages, TAD is a promising pretreatment technology for anaerobic digestion of organic solid waste. Particularly, previous studies have shown that TAD is effective in enhancing the breakdown of sewage sludge (Zhang L. et al., 2022; Jang et al., 2018; Jang et al., 2013).

The suitability of aquaculture sludge for TAD depends on its physical, chemical, and biological characteristics. Despite recent efforts to treat aquaculture sludge using anaerobic digestion processes (Siddique et al., 2021; Wu and Song, 2020; Chiumenti et al., 2020), no studies to date have evaluated the application of TAD to aquaculture sludge as a biological pretreatment technique before anaerobic digestion. A particular problem is that aquaculture sludge has a range of salinity (0.2–44.0 psu), so the effects of salinity on the TAD process must be evaluated (Kashem et al., 2023). In general, salinity can induce energetically costly stress responses in microorganisms as they balance the osmotic pressure of their cytoplasm, reducing the energy available for metabolism; this affects the microbial community and intracellular enzyme activity (Yin et al., 2022; Linaric et al., 2013). However, studies that have monitored changes in VSS reduction rates, soluble organic product concentrations, and hydrolysis effects in the TAD process under varying salinity conditions of aquaculture sludge have not been conducted. The findings from such studies could provide valuable guidance for the operation of processes combining TAD as a biological pretreatment with anaerobic digestion. The objectives of this study were to evaluate the potential of using TAD to hydrolyze fish farm sludge and to investigate how salinity of the sludge affects the hydrolysis efficiency of TAD and the microbial community structure.

## 2 Materials and methods

### 2.1 Substrate preparation

The freshwater sludge used in this study was collected from the sedimentation tank of a rainbow trout aquaculture facility located in Tongyeong, Korea. About 13 tons of fish were raised in 586 m^3^ of freshwater with a salinity of 0–0.1 psu and the fish were fed with commercial aquafeed (Daehan Feed, Korea). The sludge was processed through a 1.0-mm sieve to remove inert materials and homogenize the solids. The processed sludge was stored at −20°C until use (Table 1).

**Table 1:** Characteristics of aquaculture sludge.

Parameters	Values (average ± standard deviation)
Salinity (psu)	N.D
pH	7.44 ± 0.05
Alkalinity (g CaCO_3_/L)	0.70 ± 0.06
TSS (g/L)	87.58 ± 4.03
VSS (g/L)	36.71 ± 2.03
TCOD (g/L)	54.63 ± 0.53
SCOD (g/L)	2.03 ± 0.11
TN (g/L N)	1.21 ± 0.06
STN (g/L N)	0.19 ± 0.01
NH_4_^+^ (g/L N)	0.13 ± 0.01
NO_2_^−^ (g/L N)	N.D
NO_3_^−^ (g/L N)	N.D
PO_4_^3−^ (mg/L P)	3.11 ± 0.04
Soluble protein (g COD/L)	0.63 ± 0.03
Soluble carbohydrate (g COD/L)	0.21 ± 0.04
Total VFAs (g COD/L)	0.66 ± 0.01

“N.D”, not detected.

### 2.2 Reactor setup and operating conditions

To investigate the effects of different levels of sludge salinity on TAD performance, batch experiments were conducted using the aquaculture sludge that had been adjusted to have salinities of 0, 15, or 30 practical salinity units (psu). The salt concentration of seawater is generally 25–35 psu (He et al., 2017). These salinities were obtained using freshwater aquaculture sludge (0 psu) or by adding NaCl (99% purity, Sigma-Aldrich, USA) to the sludge to attain 15 or 30 psu. The experiments were performed in aerobic reactors that had a working volume of 800 mL, i.e., 660 mL of substrate and 140 mL of inoculum. All reactors containing substrate and inoculum were stirred at 200 rpm using inbuilt agitators while maintaining a temperature of 55 ± 1.0°C. Compressed air was continuously supplied at a rate of 2 L/min through a submerged aerator. The inoculum was obtained from excess sludge (TSS: 38.15 ± 1.41 g/L; VSS: 24.7 ± 0.71 g/L) collected from the secondary sedimentation tank of a municipal wastewater treatment plant in Tongyeong, Korea.

### 2.3 Analytical methods

Salinity was measured using a salinity refractometer (S/Mill-E, Atago, Japan), and pH was measured using a pH meter (A211, Thermo Fisher Scientific, USA). All soluble samples were filtered through a 0.45-μm polyethersulfone membrane filter (Sartorius, Germany) before analysis. TSS, VSS, TCOD, SCOD, STN, STP, and alkalinity were measured according to standard methods (Clesceri et al., 1998). Carbohydrate concentrations were determined using the phenol-sulfuric acid method (Dubois et al., 1956). Protein concentrations were measured using the Lowry-Folin method (Lowry et al., 1951). Concentrations of NH_4_^+^, NO_2_^−^, NO_3_^−^, and PO_4_^−3^ were measured using a nutrient analyzer (Quaatro39, Seal analytical, USA). Volatile fatty acids (VFAs) were quantified using a high-performance liquid chromatograph (HPLC-1100, Agilent Technology, USA) equipped with an Aminex HPX-87H column (Biorad, USA), a refractive index detector, and a diode array detector.

### 2.4 Analysis of microbial community

The microbial communities were analyzed using the Illumina iSeq 100 system (Illumina, USA) according to the manufacturer’s instructions. Samples were collected at the end of the thermophilic aerobic digestion process, and DNA from the TAD reactors (0 psu, 15 psu, and 30 psu) and the seed was extracted using the FastDNA Spin Kit for Soil (MP Biomedicals, USA) following the provided protocol. DNA amplification was performed using bacterial primers 518F (5`-CCAGCAGCCGCGGTAATACG-3`) and 805R (5`-GACT ACCAGGGTATCTAATCC-3`), targeting the V3-V4 region of the 16S rRNA gene, with an annealing temperature of 58°C. The PCR products were purified using a PCR purification kit (solgent, Korea) for library construction, then sequenced using the iSeq 100 system according to the manufacturer’s manual. The sequencing results were analyzed for taxonomic classification using the Silva rRNA database as a reference.

## 3 Results and discussion

### 3.1 Effect of salinity on the performance of TAD

**Figure 1:**
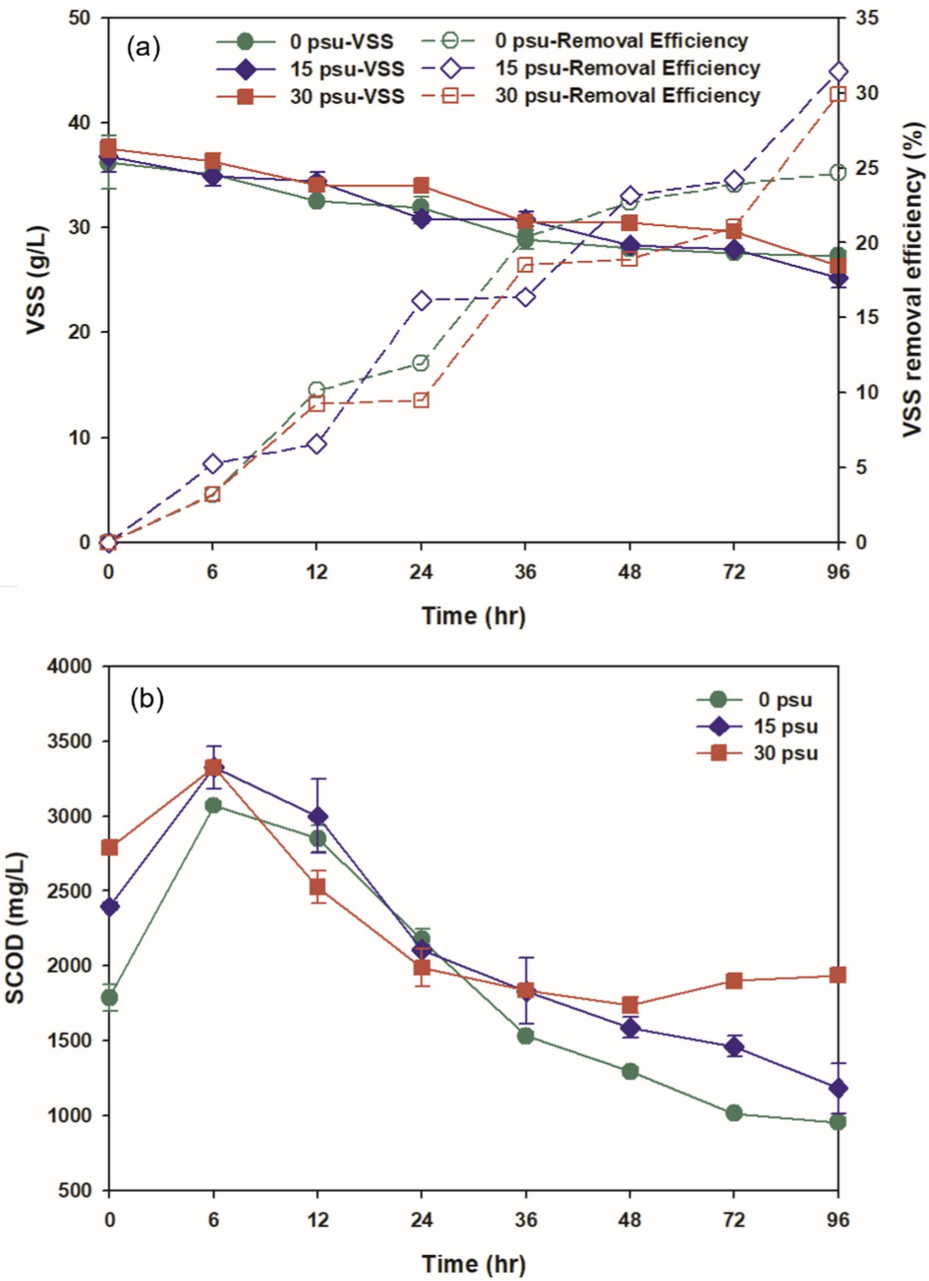
Effect of salinity levels on (a) VSS and (b) SCOD during TAD process.

VSS in all reactors gradually decreased over time, so removal efficiency increased correspondingly (Figure 1a). This trend suggests that the rapid reduction of organic solid waste was due to the active metabolism of microorganisms under thermophilic digestion conditions. Microorganisms with low resistance to high temperatures likely perished, whereas thermophilic aerobic microorganisms released heat-resistant enzymes such as protease, which enhanced the reduction of organic matter in the aquaculture sludge (Liu et al., 2018). The initial VSS concentrations were slightly higher in the 15 psu and 30 psu reactors than in the 0 psu reactor; the difference can be attributed to the salinity increasing the ionic strength of the aquaculture sludge. This increase in ionic strength compressed the electrical double layer thickness of the solid particles and reduced the zeta potential, and thereby led to coagulation and flocculation of colloidal organic matter (Linaric et al., 2013). After 96 h, the final VSS removal efficiencies in the 0 psu, 15 psu, and 30 psu reactors reached 24.64% at 0 psu, 31.43% at 15 psu, and 29.89% at 30 psu; the similarity indicates that in the first 96 h, the increase in salinity did not have a significant effect on VSS removal in the thermophilic aerobic digestion (TAD) of aquaculture sludge (Figure 1a). However, compared to the 0 psu condition, the slight increase in final VSS removal efficiencies in the 15 psu and 30 psu conditions seemed to be directly or indirectly related to the increased ionic strength of the aquaculture sludge, as mentioned above. Further studies are required to clarify these relationships. Additionally, these results showed relatively higher VSS removal rates at 96 h than the approximately 8–12% observed in TAD studies that used sewage sludge as a substrate (Jin et al., 2015; Liu et al., 2022). Compared to aquaculture sludge, 96 h of digestion time seemed insufficient to disintegrate the secondary sludge, likely due to containing more non-biodegradable or recalcitrant materials.

The solubilization and hydrolysis of organic matter are critical indicators of the overall performance and stability of TAD systems (Liu et al., 2022). The initial SCOD concentrations increased as salinity increased: from 1787.50 mg/L at 0 psu, 2393.75 mg/L at 15 psu, and 2787.50 mg/L at 30 psu. This trend may occur because increase in salinity causes increase in osmotic pressure, which leads to the release of intracellular organic constituents (Figure 1b; Li Y. et al., 2020). As digestion progressed, the SCOD concentrations in all reactors increased to maxima at 6 h: reaching 1.64, 1.39, and 1.19 times their initial values at 0 psu, 15 psu, and 30 psu, respectively (Figure 1b). The results indicated that there were significant differences (*p* < 0.05) between the salinity levels of aquaculture sludge and the SCOD concentrations. This increase in SCOD concentration in the first 6 h suggests that the release and hydrolysis of organic matter were enhanced during the thermophilic aerobic digestion process.

The hydrolysis efficiency at 6 h was 2.42% at 0 psu, 1.78% at 15 psu, and 1.04% at 30 psu; this trend indicates the hydrolysis efficiency decreases as salinity increases. This decrease in hydrolysis efficiency likely occurs because as salinity increases, the thermophilic aerobic microorganisms experience increased osmotic stress, which reduces the metabolic activity in cells that are sensitive to salt. Typically, non-halophilic microorganisms exposed to excessively high salinity environments may experience dehydration, plasmolysis, and reduced enzyme activity due to increased osmotic pressure (Wood, 2015). In this study, as salinity increased, the activity of thermophilic aerobic microorganisms gradually decreased, and this decrease in turn reduced hydrolysis efficiency. After 6 h, as digestion progressed, the SCOD concentration in all conditions steadily decreased from 12 h to 48 h, because the amount of soluble organic matter consumed by the microorganisms exceeded the amount generated by the decomposition of aquaculture sludge. At 0 psu and 15 psu, the substrate consumption rate remained higher than the hydrolysis rate even after 48 h. However, at 30 psu, the concentration of soluble organic matter did not change significantly after 48 h. This result suggests that thermophilic aerobic microorganisms exposed to prolonged osmotic stress in the elevated salt environment at 30 psu eventually underwent plasmolysis and loss of microbial activity, because they lacked mechanisms to overcome this stress.

### 3.2 Effect of salinity on release of extracellular polymeric substances

**Figure 2:**
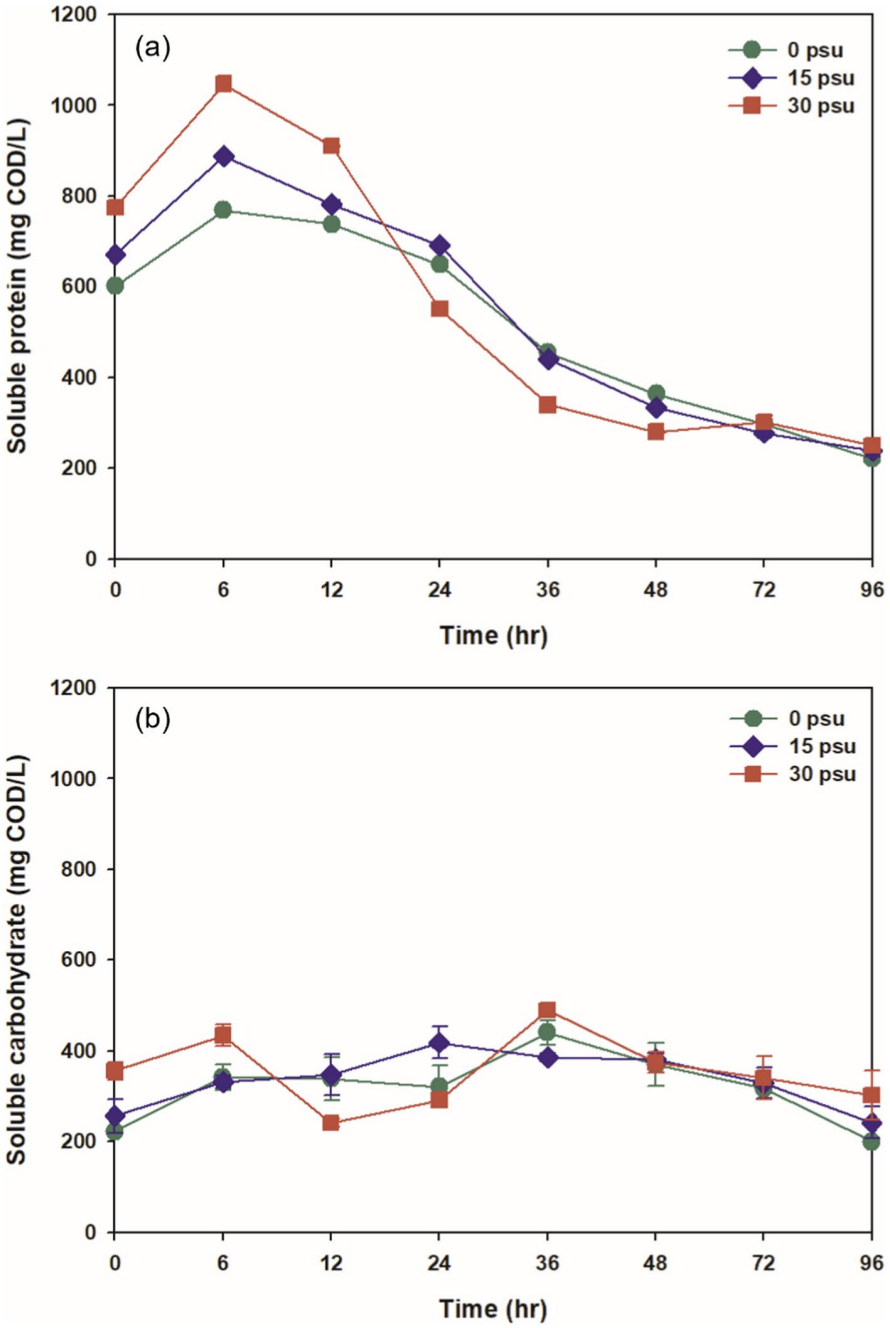
Effect of salinity levels on (a) soluble protein and (b) soluble carbohydrate during TAD process.

Extracellular polymeric substances (EPS) are polymers that are distributed on the surface of microbial cells and are produced by microorganisms during metabolic processes (Zhang M. et al., 2022). Proteins and carbohydrates are the most prominent organic matters in EPS and constitute a significant portion of the sludge’s SCOD. Initial soluble protein and soluble carbohydrate levels tend to increase as salinity increases, because the osmotic pressure resulting from high salinity stimulates non-halophilic microorganisms to produce large amounts of EPS to protect against the saline environment (Figure 2; Abbasi and Arirl, 2008).

The concentration of soluble protein is a result of hydrolysis under the presence of proteolytic enzymes. In all conditions, the concentration increased over time after 6 h of digestion as protein solubilization occurred. The increases in soluble protein concentration were 27.81% at 0 psu, 32.44% at 15 psu, and 35.14% at 30 psu (Figure 2a). The solubilization rate of proteins increased with substrate salinity after 6 h, as Na^+^ released from NaCl exchanged with divalent cations that connect EPS to the surface of microorganisms; this process promoted the release of EPS (Aloui et al., 2009; Higgins and Novak, 1997). The high salinity also appeared to facilitate the release of intracellular substances into the liquid phase. As digestion time extended beyond 6 h, soluble protein was hydrolyzed to amino acids or peptides by proteolytic enzymes, and as the rate of protein degradation surpassed the rate of protein solubilization, the concentration of soluble protein gradually decreased (Kavitha et al., 2013).

The solubilization of carbohydrates after 6 h of digestion increased by 53.61% at 0 psu, 29.25% at 15 psu, and 21.92% at 30 psu compared to the initial levels (Figure 2b). The increase in carbohydrate solubilization after 6 h decreased as substrate salinity increased; this trend was opposite to the trend in protein concentration. This opposite pattern suggests that non-halophilic and non-halotolerant microorganisms required more energy for osmotic maintenance to adapt to relatively elevated salinity; to obtain this energy, non-halophilic and non-halotolerant microorganisms increased their consumption of soluble carbohydrates so the rate of hydrolysis of carbohydrates decreased (Lay et al., 2010). As the digestion time continued to 36 h, the concentration of soluble carbohydrates fluctuated due to varying relative rates of substrate utilization and hydrolysis by microorganisms under each condition. In all conditions, the concentration of carbohydrates steadily decreased after 36 h as they were hydrolyzed to simple sugars.

### 3.3 Variations in pH, alkalinity, and volatile fatty acids

**Figure 3:**
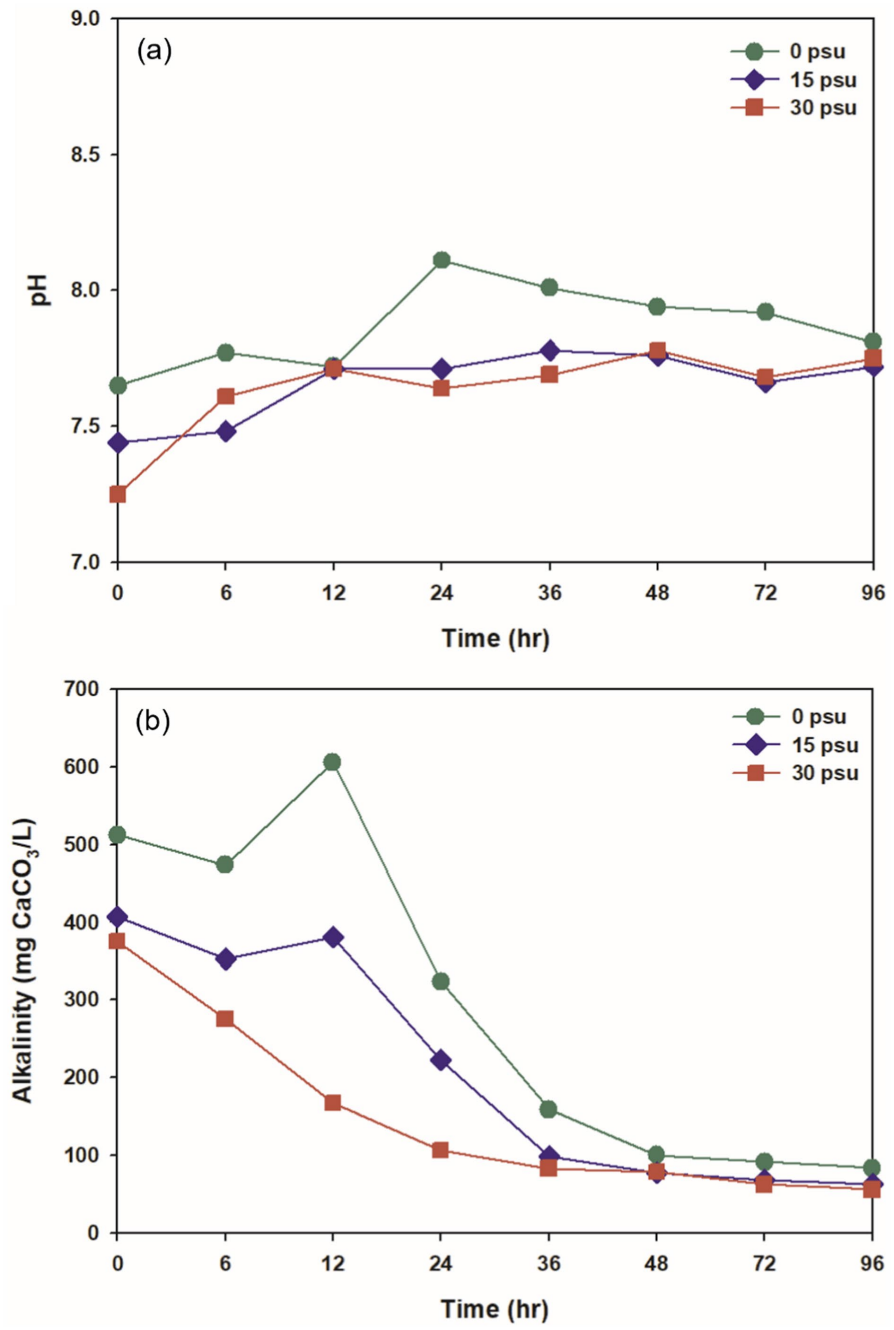
Variations at salinity levels during TAD process: (a) pH and (b) alkalinity.

The pH during thermophilic aerobic digestion is influenced by several factors, including VFAs, nitrogen compounds, temperature, and the hydrolysis of organic matter (Liu et al., 2022). Throughout the digestion period, the pH remained slightly alkaline (7.25 to 8.11) at all psu (Figure 3a). The initial pH decreased slightly as salinity increased, to 7.65 at 0 psu, 7.44 at 15 psu, and 7.25 at 30 psu. During the digestion period, the pH remained higher at 0 psu than at 15 and 30 psu. This difference is likely due to the difference in ionization fractions of total inorganic carbon species between saline water and freshwater: more total inorganic carbon ionizes to CO_2_ in saline conditions than freshwater conditions, so the degree of pH increase caused by CO_2_ stripping during digestion is affected (Moran, 2010).

**Figure 4:**
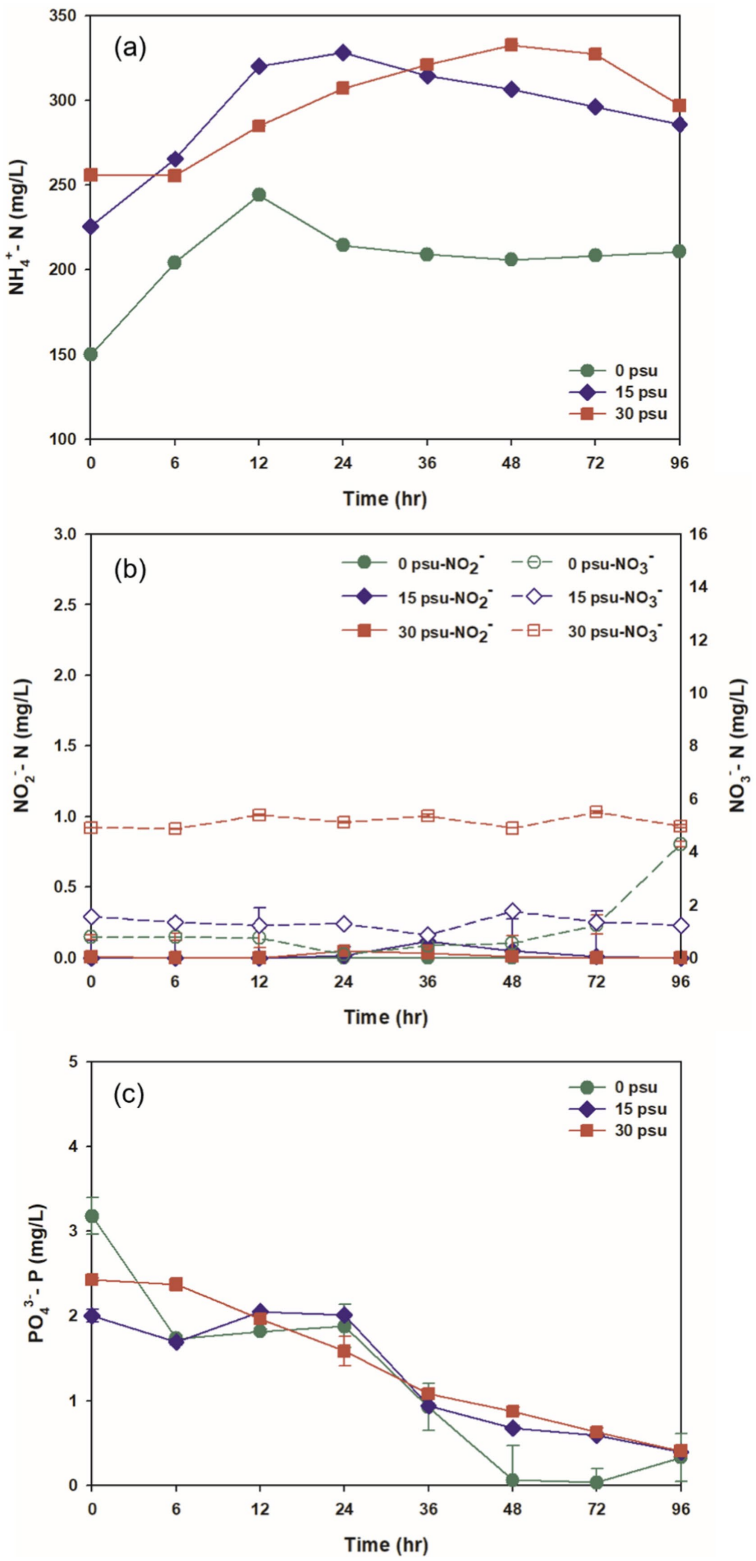
Variations at salinity levels during TAD process: (a) NH_4_^+^, (b) NO_2_^−^ and NO_3_^−^, (c) PO_4_^−3^.

**Figure 5:**
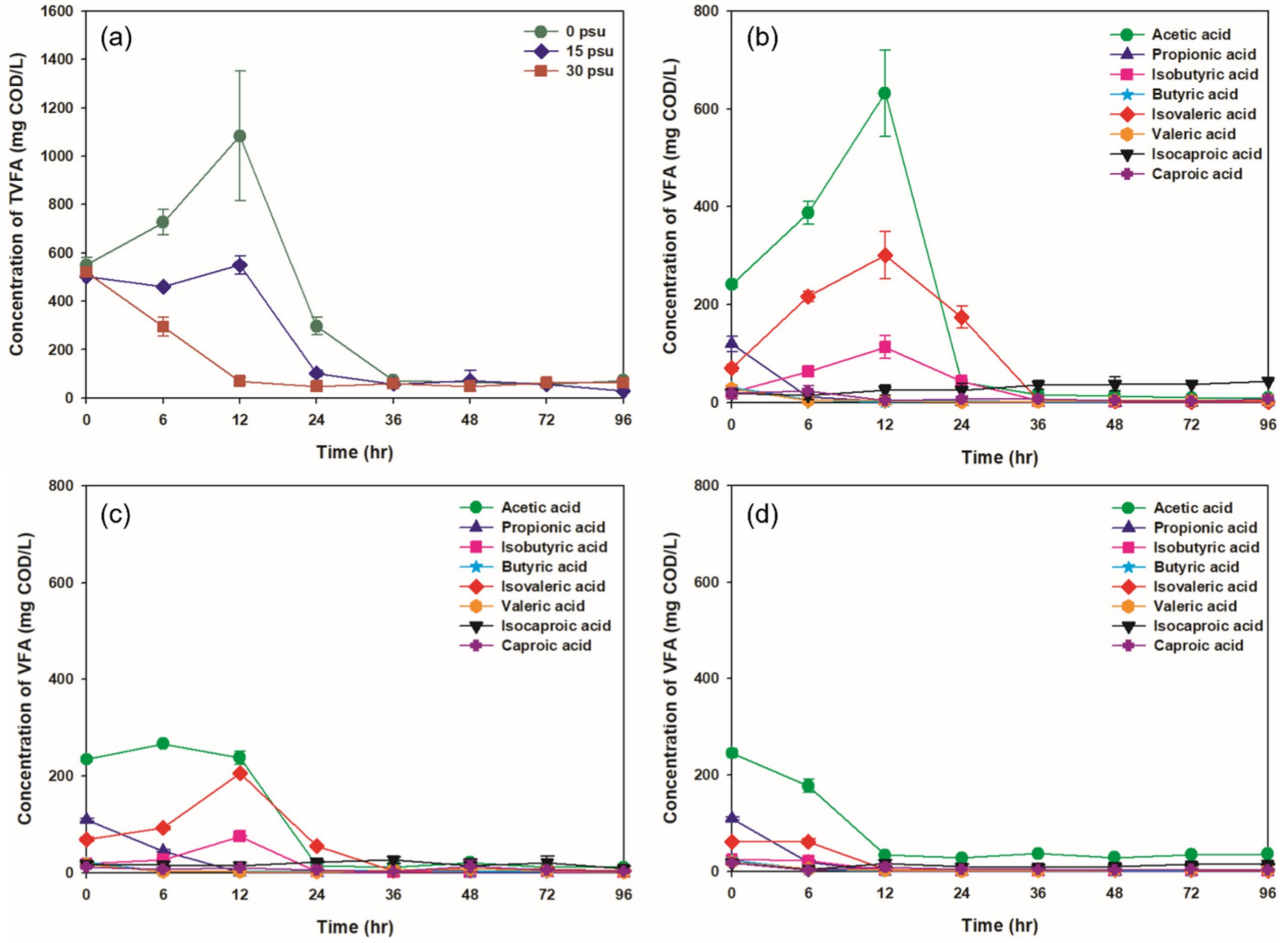
Variations at salinity levels during TAD process: (a) TVFAs concentration under all psu conditions, (b) VFAs composition at 0 psu, (c) VFAs composition at 15 psu, (d) VFAs composition at 30 psu.

Initial alkalinity decreased as salinity increased. At 0 psu, the initial alkalinity was 512.02 mg CaCO_3_/L, and increased to a maximum after 12 h due to the sharp increase in NH_4_^+^ generated during the digestion process (Figures 3b, 4a). At 15 psu, the initial alkalinity was 407.42 mg CaCO_3_/L, and this value was the same after 12 h. At 30 psu, the initial alkalinity was 375.63 mg CaCO_3_/L, and decreased continuously for the first 12 h. Despite the increase in ammonium concentration at 15 psu and 30 psu during the first 12 h of thermophilic aerobic digestion, alkalinity tended to decrease as salinity increased. This result may be a result of increased ionization of total inorganic carbon into CO_2_ as salinity increased; this process would decrease the alkalinity. Additionally, the heightened osmotic pressure from increased salinity likely caused dehydration and plasmolysis of bacteria that produce alkalinity, and thereby inhibited their activity and resulted in a decrease in alkalinity (Guo et al., 2020; Cho et al., 2013). At all salinities, alkalinity decreased over time; this trend is attributed to the stripping of CO_2_ and NH_3_ by consistent aeration, and to neutralization of organic acids produced by biological activity (Figures 3b, 4a, 5).

VFAs are intermediate products formed during the thermophilic aerobic digestion process; they can be generated by partial oxidation and solubilization of substrates. At 0 psu, the concentration of total VFAs (TVFAs) peaked after 12 h, but as digestion continued, the accumulated TVFAs were broken down by thermophilic aerobic microorganisms so the VFA concentration decreased (Figure 5A; Liu et al., 2012). In contrast, at 15 psu, the TVFA concentration remained stable for the first 12 h before decreasing, whereas at 30 psu, it declined continuously as digestion progressed. Generally, the accumulation of VFAs in thermophilic aerobic digestion occurs when the oxygen-transfer rate to the microorganisms is lower than their oxygen demand. However, in this study, increased salinity in the aquaculture sludge seemed to inhibit the accumulation of VFAs (Chu et al., 1997).

The predominant VFAs at 6 h and 12 h at all salinities were acetic, iso-valeric, and iso-butyric acids, with acetic acid the most common. The metabolic pathways for production of specific VFAs vary depending on the substrate. Breakdown of carbohydrates produces mainly acetic and propionic acids, whereas breakdown of proteins produces mainly acetic, butyric, and valeric acids (Xu et al., 2013). Other VFAs can be converted to acetic acid by microbial metabolism. The fact that all of these processes produce acetic acid explains why it was the most common VFA at 6 h and 12 h.

### 3.4 Variations in nitrogen components and phosphate

Degradation of proteins and subsequent lysis of thermally sensitive cells during thermophilic aerobic digestion lead to release of nitrogenous compounds in the reactor. Ammonium, in particular, is released into the supernatant following the deamination of peptides and of amino acid products of protein hydrolysis (Liu et al., 2012). The initial concentrations of ammonium nitrogen (NH_4_^+^-N) were 149.94 mg/L at 0 psu, 225.53 mg/L at 15 psu, and 256.04 mg/L at 30 psu. The increase with salinity indicates that increased salinity corresponds to elevated initial NH_4_^+^-N concentrations likely because osmotic pressure increased as salinity increased, and therefore induced increased release of intracellular nitrogen components (Li X. et al., 2020; Figure 4a).

As digestion progressed, NH_4_^+^-N concentrations increased at all salinities, and peaked at 12 h. This increase was attributed to the release of NH_4_^+^-N by breakdown of proteins and lysis of thermally-sensitive microorganisms. At 0 psu, after 12 h, the NH_4_^+^-N concentration under the 0 psu condition began to decrease, likely due to the combined effects of microbial metabolism by thermophilic aerobic microorganisms and ammonia stripping during aeration, then protein hydrolysis, microbial metabolic uptake, and ammonia stripping seem to have reached a balance. In contrast, at 15 psu and 30 psu, NH_4_^+^-N concentrations remained maintained consistently higher than at 0 psu throughout the digestion period. This result may occur because compared to the 0 psu case, these reactors had lower pH, which could inhibit ammonia stripping (Figure 3a). Nitrification and denitrification are inhibited at temperatures >40°C, so the concentrations of nitrite nitrogen and nitrate nitrogen remained >0.2 mg/L and > 6.0 mg/L respectively, across all salinity conditions, and did not change significantly (Figure 4b; Jin et al., 2015).

The concentration of PO_4_^−3^ decreased consistently at all salinities for the first 96 h. This trend suggests that phosphate was more extensively utilized for adenosine triphosphate (ATP) synthesis by oxidative phosphorylation in thermophilic aerobic microorganisms, rather than being released into the environment by the lysis of thermally-sensitive microbial cells (Figure 4c; Jin, 2018).

### 3.5 Effect of salinity on microbial community composition

**Figure 6:**
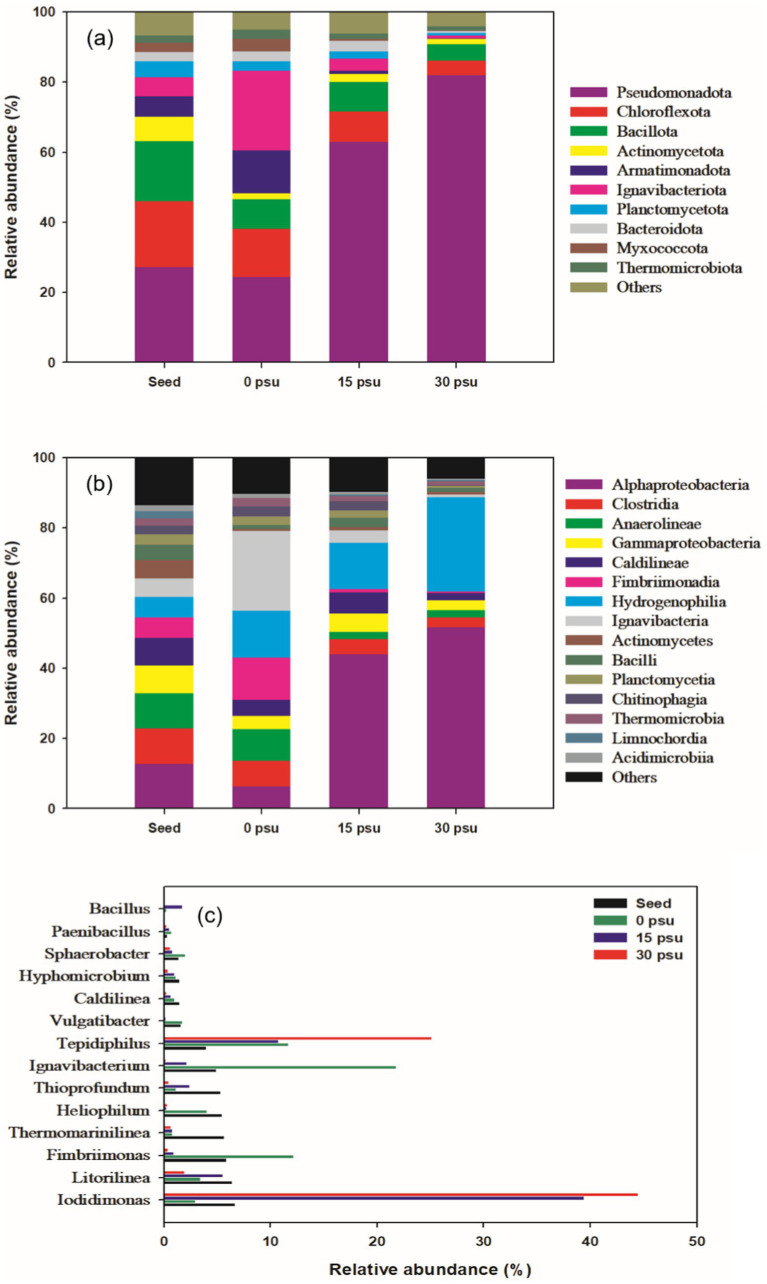
Comparison of bacterial community changes at different salinity levels during TAD process: (a) phylum level, (b) class level, (c) genus level.

Salinity affected the microbial community structure. At all salinities, the predominant phyla observed were Pseudomonadota, Chloroflexota, and Bacillota. The relative abundance of Pseudomonadota increased as salinity increased (Figure 6a). Pseudomonadota is associated with hydrolysis, inducing cell lysis that releases intracellular organic matter, and with breaking down organic compounds such as proteins and carbohydrates (Gao et al., 2022). Additionally, Pseudomonadota is known to dominate in saline environments (Chen et al., 2022). The relative abundances of Chloroflexota and Bacillota gradually decreased as salinity increased. Chloroflexota contributes to polysaccharide degradation and hydrolysis by producing extracellular enzymes. Bacillota can degrade a variety of substrates such as cellulose, proteins, lignin, and lipids by producing extracellular enzymes (Petriglieri et al., 2023; Liu et al., 2022).

Phyla Armatimonadota and Ignavibacteriota were relatively abundant at 0 psu, but their relativae abundances declined steeply as salinity increased. Armatimonadota has been reported to have a strong ability to disrupt branched or amorphous polysaccharides. Ignavibacteriota has strong hydrolytic abilities across a broad range of substrates (Zamorano-López et al., 2019; Ping et al., 2018). Therefore, at 0 psu, Armatimonadota and Ignavibacteriota likely contributed to the sludge solubilization, but their adaptability decreased at salinities ≥15 psu.

At the class level, the dominant groups varied according to salinity. At 0 psu, the dominant classes were Ignavibacteria, Fimbriimonadia, Anaerolineae, and Hydrogenophilia, but at 15 psu and 30 psu, the relative abundances of these classes, except for Hydrogenophilia, significantly decreased (Figure 6b). Ignavibacteria inhabits thermophilic aquatic environments, in which it contributes to degradation of organic matter. Anaerolineae is also primarily involved in degradation of organic matter (Podosokorskaya et al., 2023; Yun et al., 2021). Fimbriimonadia is often found in environments such as marine sediment or hot-spring water, although its physiological function is not well understood (Li et al., 2024).

As salinity increased, the relative abundances of Hydrogenophilia and Alphaproteobacteria increased. Hydrogenophilia has an obligate respiratory metabolism; the group utilizes oxygen at high temperatures and can grow heterotrophically using organic acids (Boden et al., 2017; Slobodkina et al., 2020). Alphaproteobacteria can degrade organic matter even under high salinity conditions, and due to its high proteolytic activity, likely contributed to the increased NH_4_^+^ concentrations at 15 psu and 30 psu compared to 0 psu (Figure 4a; Hou and Zhu, 2024).

At 0 psu, bacterial groups such as Ignavibacteria, Fimbriimonadia, and Hydrogenophilia appeared to have contributed significantly to the decomposition of organic matter in the sludge, whereas at 15 psu and 30 psu, bacterial groups such as Alphaproteobacteria and Hydrogenophilia seemed to have influenced the digestion efficiency.

Further analysis of the community compositions at the genus level revealed notable differences in the relative abundances of dominant bacteria between 0 psu and 30 psu (Figure 6c). Particularly, the relative abundance of genera *Iodidimonas* and *Tepidiphilus* increased significantly as salinity increased. *Iodidimonas* is a halophilic, chemoorganotrophic bacterium known for its antimicrobial and sporicidal properties against various bacterial strains and spores by use of extracellular enzymes (Acharya et al., 2023; Ishii and Wakai, 2020; Iino et al., 2016). In this study, the halophilic and antimicrobial characteristics of *Iodidimonas* likely made it the dominant genus under high salinity conditions of 15 psu and 30 psu. *Tepidiphilus* thrives in extreme environments and can grow in conditions with up to 8% NaCl, as well as in high-temperature environments such as hot springs or geothermal areas (Wang et al., 2020). This genus can grow heterotrophically by utilizing various types of organic acids (Dessi et al., 2019).

The relative abundances of genera *Ignavibacterium*, *Vulgatibacter*, *Hyphomicrobium*, *Caldilinea*, *Sphaerobacter*, *Fimbriimonas*, *Heliophilum*, and *Paenibacillus* decreased as salinity increased. These genera can degrade macromolecular organics by producing hydrolases (Wang et al., 2021; Ding et al., 2022; Wu et al., 2022; Ji et al., 2020; Zakaria et al., 2020; Li Y. et al., 2020). *Caldilinea* is a thermophilic genus that achieves high levels of exoenzyme expression, and therefore can degrade macromolecules (Yang et al., 2015; Li et al., 2015). *Sphaerobacter* can break down carbohydrates and cellulose (Yang et al., 2023). *Ignavibacterium* can contribute to the decomposition of complex polymers such as cellulose, hemicellulose, and chitin (Bei et al., 2021). *Vulgatibacter* is an obligately aerobic organotroph bacterium that can degrade lignocellulosic materials such as cellulose, hemicellulose, starch, and lignin (Song C. et al., 2021). *Hyphomicrobium*, which is abundant in activated sludge from biological wastewater treatment, has the ability to decompose organic matter (Wang et al., 2022). *Fimbriimonas* is a well known hydrolytic bacterium that can use various carbon sources (Ji et al., 2020; Feng et al., 2016). *Heliophilum* can assimilate acetate, lactate, and pyruvate during photoheterotrophic growth (Bender et al., 2022). *Paenibacillus* secretes protease during aerobic digestion, thereby promoting the decomposition and transformation of organic matter (Li X. et al., 2020).

Genera *Litorilinea* and *Bacillus* showed relatively high abundance only at 15 psu. *Litoilinea* is a thermophilic, aerobic, and chemo-organotrophic bacterium that can promote protein hydrolysis of substrates (Kale et al., 2013; Zhang A. et al., 2022). Members of *Bacillus* can produce cellulase, hemicellulase, ligninase, amylase, protease, and pectinase, which likely contributed to the hydrolysis of aquaculture sludge at 15 psu (Song T. et al., 2021).

Most of the key genera that contribute to hydrolysis tended to decrease as salinity increased. This trend was mainly attributed to the increased salinity. The increase in salinity during thermophilic aerobic digestion of aquaculture sludge altered the microbial community structure, which in turn influenced the efficiency of organic matter hydrolysis.

## 4 Conclusion

The hydrolytic performance of TAD and the associated microbial communities were significantly influenced by the salinity levels of aquaculture sludge. As the salinity of sludge increased, the hydrolytic efficiency of TAD decreased. These findings suggest that in aquaculture sludge digestion systems, the effectiveness of TAD as a biological pretreatment technology before anaerobic digestion can be hindered by increased salinity levels, which limit hydrolytic enrichment. TAD can be an attractive pretreatment technology before anaerobic digestion for large-scale biogas production using aquaculture sludge. However, information on the influence of various salinity levels in aquaculture sludge on hydrolysis efficiency still remains limited, though such information is crucial for maximizing the hydrolysis efficiency of TAD. Further studies are required to determine the effects of a broad range of salinity levels on hydrolysis efficiency during continuous operation, which will be essential for optimizing the practical application of TAD with aquaculture sludge.

## Data Availability

The datasets presented in this study can be found in online repositories. The names of the repository/repositories and accession number(s) can be found in the article/supplementary material.
